# Implications of gut microbiota dysbiosis and fecal metabolite changes in psychologically stressed mice

**DOI:** 10.3389/fmicb.2023.1124454

**Published:** 2023-05-05

**Authors:** Yi Zhang, Jing Zhang, Jianmin Wu, Qinwen Zhu, Changrong Chen, Yanning Li

**Affiliations:** ^1^School of Basic Medicine, Gannan Medical University, Ganzhou, China; ^2^Department of Stomatology, The First Affiliated Hospital of Gannan Medical University, Ganzhou, China

**Keywords:** psychological stress, affective disorders, fecal microbiota transplantation, gut microbiome, fecal metabolomics

## Abstract

**Introduction:**

Psychological stress can induce affective disorders. Gut microbiota plays a vital role in emotional function regulation; however, the association between gut microbiota and psychological stress is poorly understood. We investigated effects of psychological stress on the gut microbiome and fecal metabolites and assessed the relationship between affective disorder behavior and altered fecal microbiota.

**Methods:**

A psychological stress model was established in C57BL/6J mice using a communication box. Sucrose preference test, forced swim test, and open field test helped assess anxiety- and depression-like behaviors. Fecal microbiota transplantation (FMT) was conducted using fecal samples from stressed and non-stressed mice. Moreover, 16S rRNA gene sequencing and untargeted metabolomics were performed

**Results:**

After stress exposure for 14 days, a significant increase in anxiety- and depression-like behaviors was observed. FMT of “affective disorder microbiota” from psychologically stressed mice increased stress sensitivity relative to FMT of “normal microbiota” from non-stressed mice. 16S rRNA gene sequencing revealed decreased abundance of *Bacteroides*, *Alistipes*, and *Lactobacillus* and increased abundance of Parasutterella and *Rikenellaceae_RC9_gut_group* in stressed mice; furthermore, stressed mice showed differential metabolite profiles. KEGG pathway analysis indicated that differential metabolites were chiefly involved in the downregulated pathways of α-linolenic acid metabolism, taste transduction, and galactose metabolism. *Alistipes* and *Bacteroides* were mainly positively correlated and *Parasutterella* was mainly negatively correlated with diverse metabolites.

**Discussion:**

Our findings suggest that gut microbiome dysbiosis contributes to affective disorder development in response to psychological stress.

## Highlights

1. Psychological stress induced weight loss and depression-and anxiety-like behavior.
2. FMT of microbiota derived from stressed mice increased stress sensitivity.
3. Stressed and non-stressed mice exhibited distinct metabolite profiles.
4. Gut microbiome dysbiosis contributes to affective disorder development in response to psychological stress.

## 1. Introduction

Affective disorders are highly prevalent across the world. Prolonged or repeated exposure to physical and psychological stress is a major risk factor for their development (Mahar et al., 2014; Deng et al., 2016). The mechanisms underlying affective disorders under conditions of stress have not been completely elucidated. Gut microbiota has been reported to play a crucial role in regulating emotional function. The human intestine is an extremely complex ecosystem, harboring nearly 100 trillion bacteria. The interactions between microbiota and intestinal epithelium can indirectly cause physiological changes in the brain, and also affect mood and behavior (Grochowska et al., 2018; Generoso et al., 2021). Further, in patients with affective disorders and animal models of psychiatric diseases, disturbances in gut microbiota have been identified (Rogers et al., 2016; Wong et al., 2016; Dickerson et al., 2017). It is reported that changes in *Prevotella* and *Klebsiella* proportions in fecal microbial communities were consistent with Hamilton depression rating scale in patients with major depressive disorder (Lin et al., 2017). Gut microbiota dysbiosis has also been found to induce depression-like symptoms in normal mice (Zhang et al., 2019).

The gut microbiome is sensitive to stressors (Bharwani et al., 2016), and activation of the hypothalamus–pituitary–adrenal axis (HPA axis) can evidently influence population levels (Rios et al., 2017). An increasing number of studies have reported that stress not only increases intestinal permeability but also promotes bacterial infection and invasion, leading to changes in intestinal microbiota composition (Molina-Torres et al., 2019). Stressors can alter intestinal microbial communities, eventually contributing to stressor-induced changes in immune function, neurodevelopment, and behavior (Mackos et al., 2016). Previous study has found *Oscillospira*, *Lactobacillus*, *Akkermansia*, and *Anaeroplasma* to be the most affected genera between control and post-traumatic stress disorder-exposed mice (Gautam et al., 2018). In addition, multiple physical stress models induced the dysbiosis of gut microbiota (Gong et al., 2021; Liu et al., 2022), and the alterations of lipid and amino acid metabolism were their fecal metabolome features (Liu et al., 2022).

Psychological and physical stress differentially impact the cognitive, emotional, and physical functions of animals (Liu et al., 2018). To evoke pure psychological stress and to avoid interference by physical stress, in this study, we established a psychological stress model in C57BL/6 J mice using communication box paradigm. Our core objectives were to investigate the effects of psychological stress on gut microbiome and fecal metabolite profile, and to explore the association between affective disorders and altered fecal microbiota under psychological stress.

## 2. Materials and methods

### 2.1. Animals

Adult male C57BL/6 J mice (age: 2 months, weight: 21–25 g) were group housed in individually ventilated cages under a 12-h light/dark cycle (lights were turned off at 6: 00 p.m.). The animals had *ad libitum* access to food and water. All animal procedures were performed according to the National Institutes of Health Guide for the Care and Use of Laboratory Animals, and all study protocols were approved by the Institutional Animal Care and Use Committee of Gannan Medical University.

### 2.2. Psychological stress paradigm

**Figure 1:**
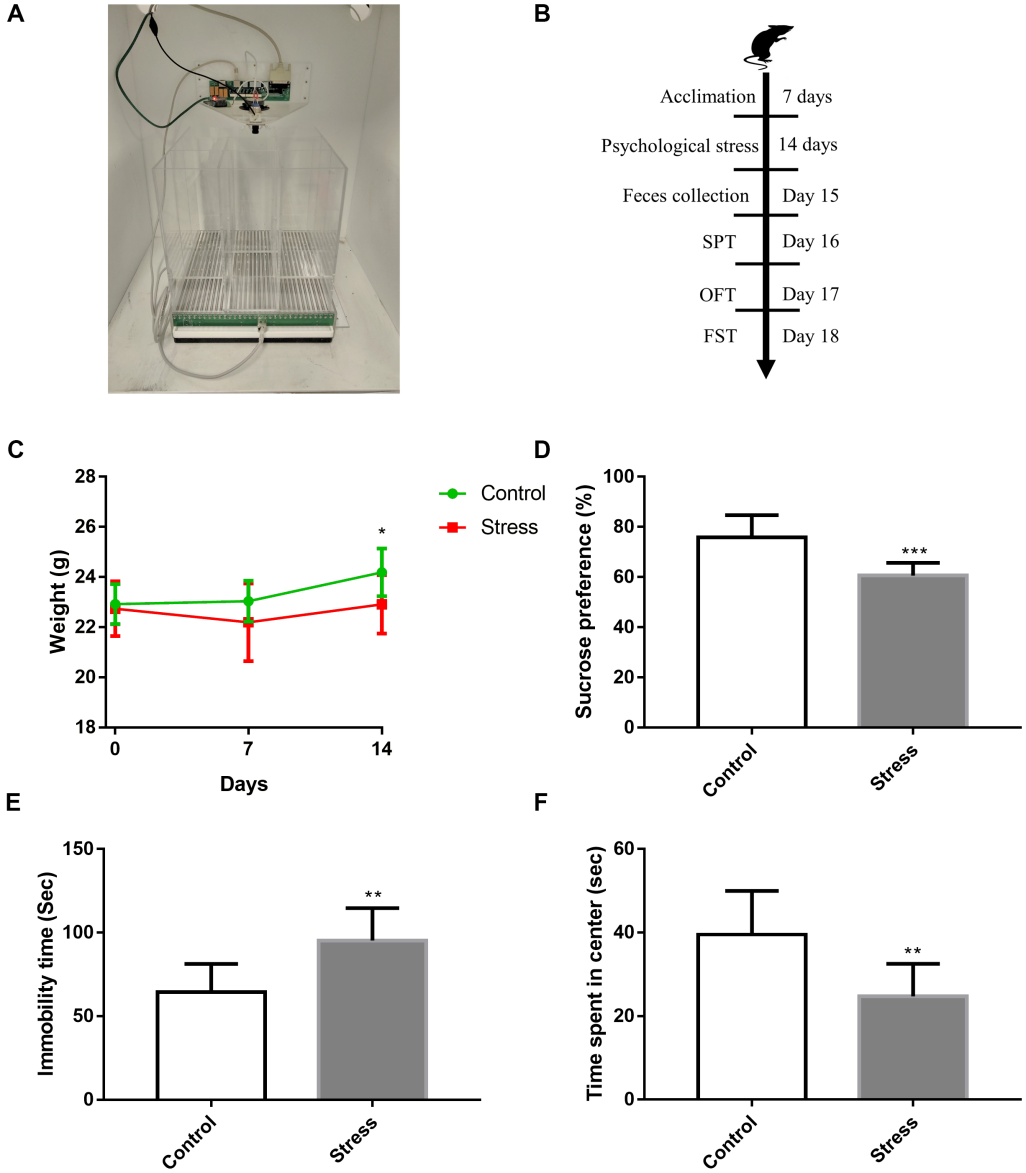
Effects of psychological stress on affective disorder behavior. **(A)** Construction of the modeling device. **(B)** Timeline of psychological stress and behavioral procedures. **(C)** Weekly weight of mice [*F*_(1,28)_ = 4.815, *p* = 0.0367]. **(D)** Sucrose preference, as evaluated via SPT (*t* = 4.664, *p* < 0.001). **(E)** Immobility time, as measured by FST (*t* = 3.782, *p* = 0.0014). **(F)** Time spent in the center of the open field (*t* = 3.585, *p* = 0.0021). Values represent mean ± SD, C: *n* = 15/group; D–F: *n* = 10/group. Student’s *t*-test was used to compare differences. The weight of mice was statistically evaluated using repeated measures ANOVA. (*p < 0.05, **p < 0.01, ***p < 0.001 vs. the control group).

Based on a previously reported protocol (Murata et al., 2017; Li et al., 2021), a communication box system was used for inducing psychological stress. The apparatus included a transparent box (30 cm × 30 cm × 30 cm), which was separated by transparent plexiglass plates into nine compartments, with several small holes (2 mm in diameter) carved on the plates between compartments (Figure 1A). The bottom of the communication box was equipped with a grid floor composed of stainless-steel rods (2.5 mm in diameter and spaced 5-mm apart). Plastic insulator plates were placed on the grid floors of four compartments other than the center and four corners. To expose mice to psychological stress, five mice were individually placed in foot-shock compartments, and four mice were individually placed in psychological stress compartments that were covered with a plastic insulator to avert electric shock. Psychologically stressed mice received visual, auditory, and olfactory emotional stimuli (such as screaming, jumping, and evacuation) from mice receiving electric foot shock. The animals were exposed to psychological stress at the same time every day, and they received 0.5–1 mA electric current (10-s duration with 50-s interval) via a shock generator for 30 min each day for 14 days. Mice receiving electric shock were replaced timely to avoid adaptation. Sham-treated controls were placed in compartments, similar to the aforementioned setup, but did not receive any electric stimuli.

### 2.3. Sucrose preference test

The sucrose preference test (SPT) was used to assess anhedonia, which is the core symptom of depression, as previously described (Li et al., 2021). Mice were fed in a single cage; on the first day, they were given two bottles of 1% sucrose. On the second day, mice were given a bottle of water and a bottle of 1% sucrose, and on the third day, all mice were deprived of water for 24 h. On the fourth day, they were given a bottle of 1% sucrose and a bottle of water for 1 h, and the consumption of sucrose and water was recorded. Mice were put back to group housed cages after test. Sucrose preference percentage was calculated using this formula: sucrose solution consumption/ (sucrose consumption + water consumption) × 100%.

### 2.4. Forced swim test

The forced swim test (FST) was used to evaluate depressive-associated behavior in animals (Cao et al., 2013). Each mouse was placed in a transparent plexiglass hollow cylinder (height: 25 cm, diameter: 10 cm). The depth of water (23°C–25°C) was approximately 20 cm, and mice were unable to touch the bottom for support. During the experiment, mice were gently placed in water and allowed to freely swim for 6 min. Cumulative immobility time (immobility was defined as the absence of active struggle, only with the body floating in water) was recorded for the last 4 min.

### 2.5. Open field test

Open field test (OFT) was performed to evaluate anxiety-like behavior, as previously described (Li et al., 2017), using a four-walled black plastic box (40 cm × 40 cm × 30 cm) with a white bottom and no top. The squares adjacent to the walls were designated as “periphery”; all remaining ones were designated as “center” (23 cm × 23 cm). Mice were placed in a corner of the box, and a video camera was used to record their behavioral performance for 10 min. The time spent by mice in the center of the open field was calculated.

### 2.6. Fecal microbiota transplantation

Fecal sample preparation and fecal microbiota transplantation (FMT) were performed as previously described (Liu et al., 2021). Briefly, fecal samples were collected from psychologically stressed and non-stressed (i.e., control) mice (*n* = 10 each group) 24 h after psychological stress paradigm, immediately frozen, and stored at −80°C. The samples (150 mg) were then resuspended in 1 ml sterile PBS, mixed, and centrifuged at 3,000× *g*, and the supernatant thus obtained was collected, and 10% glycerin was added to the solution. Before FMT, native gut microbiota was eliminated by administering antibiotics (100 mg/kg vancomycin, 200 mg/kg neomycin sulfate, 200 mg/kg metronidazole, and 200 mg/kg ampicillin, Shanghai Macklin Biochemical Co., Ltd., China) for 5 days. Most antibiotics used were non-absorbable, suitable for the gut. Subsequently, the microbiota supernatant (0.1 ml) was administered to these microbiota-depleted mice by gavaging for 14 days. Mice were sham-treated or exposed to psychological stress until the end of FMT.

### 2.7. 16S rRNA gene sequencing

Fresh fecal samples were collected from mice (*n* = 6 each group), and DNA was extracted using the cetyltrimethyl ammonium bromide/sodium dodecyl sulfate (CTAB/SDS) method. DNA concentration and purity were monitored on 1% agarose gels. DNA samples were then diluted to 1 ng/μL with sterile water. The V3 − V4 hypervariable regions of the 16S rRNA gene were amplified by performing PCR with specific primers (338F, 5′-ACTCCTACGGGAGGCAGCAG-3′ and 806R, 5′-GGACTACHVGGGTWTCTAAT-3′) and barcodes. Amplicons were excised from 2% agarose gels and purified using the QIAquick Gel Extraction Kit (Qiagen, Germany). Sequencing libraries were generated with the NEBNext® Ultra™ IIDNA Library Prep Kit (New England Biolabs, United States) and sequenced on an Illumina NovaSeq platform (Illumina, United States), which led to the generation of 250-bp paired-end reads. The resultant sequences were merged with FLASH v1.2.11 and quality filtered with FASTP v0.20.0; chimera sequences were detected with Vsearch v2.15.0 and removed. Denoising was performed with DADA2 in QIIME2 (vQIIME2-202,006) to obtain initial amplicon sequence variants (Callahan et al., 2016). Species annotation and multiple sequence alignment were performed using QIIME2 to study phylogenetic relationship of each amplicon sequence variant and to assess differences in dominant species among different groups.

### 2.8. Untargeted metabolomics

Fecal samples (100 mg, *n* = 5 each group) were individually ground with liquid nitrogen, and the homogenate was thoroughly mixed in prechilled 80% methanol by vortexing. The samples were then incubated on ice for 5 min and centrifuged at 15,000 g and 4°C for 20 min. The supernatant was analyzed by UHPLC–MS/MS, which was performed on a Vanquish UHPLC system (Thermo Fisher, Germany) coupled with an Orbitrap Q Exactive™ HF-X mass spectrometer (Thermo Fisher, Germany). The samples were injected onto a Hypersil Gold column (100 mm × 2.1 mm, 1.9 μm) using a 17-min linear gradient at a flow rate of 0.2 mL/min. The Q Exactive™ HF-X mass spectrometer was operated in positive/negative polarity mode with spray voltage of 3.5 kV, capillary temperature of 320°C, sheath gas flow rate of 35 psi, aux gas flow rate of 10 l/min, aux gas heater temperature of 350°C. Raw data generated by UHPLC–MS/MS were processed using Compound Discoverer 3.1, peak alignment, peak picking, and quantitation were performed for each metabolite. Normalized data were used for molecular formula prediction based on additive ions, molecular ion peaks, and fragment ions. Subsequently, to obtain accurate qualitative and relative quantitative results, the peaks were matched with mzCloud, mzVault, and MassList. Partial least squares discriminant analysis (PLS-DA) was performed using metaX (Wen et al., 2017). Metabolites with VIP > 1, *p* < 0.05, and fold change >1.2 or < 0.833 were regarded to be differentially expressed. Their functions and metabolic pathways were studied using the Kyoto Encyclopedia of Genes and Genomes (KEGG) database.

### 2.9. Statistical analysis

Values represent mean ± SD. Student’s t-test or two-way analysis of variance with Tukey’s post-hoc test were employed to compare differences among groups. GraphPad Prism 7.0 (San Diego, CA, United States) was used for statistical analysis. *p* < 0.05 indicated statistical significance.

## 3. Results

### 3.1. Psychological stress induced weight loss and affective disorder behaviors

After psychological stress exposure for 14 days, the body weight of mice was recorded, and behavioral tests were performed (Figure 1B). We found stressed mice had significantly lower mean body weight than non-stressed mice (Figure 1C). Depression-like behavior was evaluated via SPT and FST; relative to non-stressed mice, stressed mice displayed a significant decrease in sucrose consumption (Figure 1D) and longer immobility time (Figure 1E). Further, anxiety-like behavior was evaluated via OFT; in comparison to non-stressed mice, stressed mice spent significantly lesser time in the center of the open field (Figure 1F). Collectively, these data indicated that psychological stress induced weight loss and affective disorder behaviors in mice.

### 3.2. Psychological stress induced gut microbiota dysbiosis

**Figure 2:**
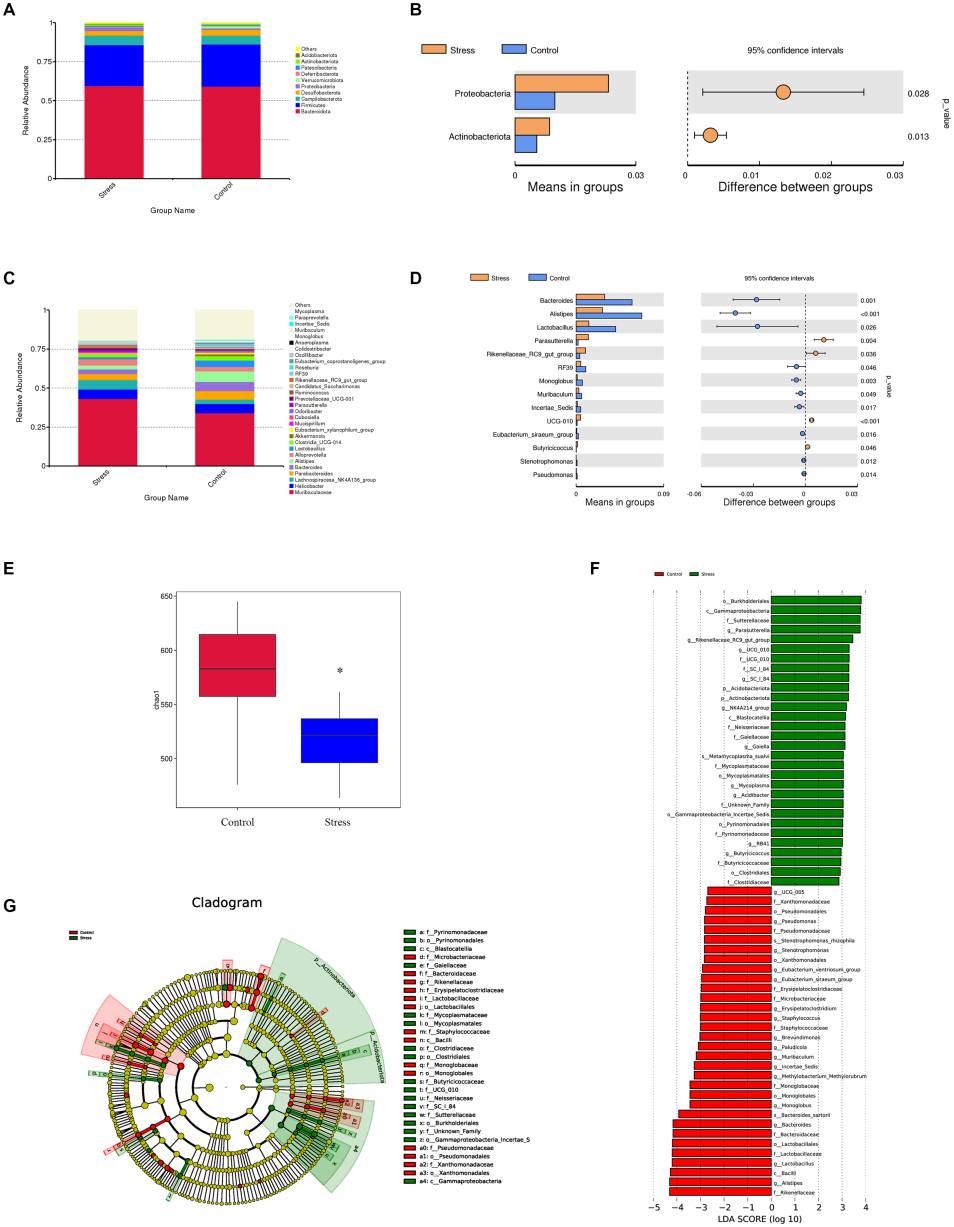
Impact of psychological stress on gut microbiota composition (*n* = 6/group). **(A)** Abundance of gut microbiota at the phylum level. **(B)** Significantly different phyla between the groups (*t*-test). **(C)** Abundance of gut microbiota at the genus level. **(D)** Significantly different genera between the groups (*t*-test). For panels **(B,D)**, the left panel shows abundance of differential species and the right panel shows 95% confidence intervals. **(E)** Chao1 index of fecal samples from control or stressed mice (*p* = 0.0345). **(F)** Differentially expressed taxa identified by LEfSe analysis between the groups (The threshold of the logarithmic linear discriminant analysis score was >2.0). **(G)** Cladogram from LEfSe analysis, representing the classification level from phyla to genera.

To investigate whether psychological stress induces obvious changes in gut microbiota, 16S rRNA genes of fecal microbiota were analyzed. We found that gut microbiota composition was significantly altered in stressed mice. The most abundant phyla were *Bacteroidota*, *Firmicutes*, and *Campilobacterota* (Figure 2A), and the abundance of *Proteobacteria* and *Actinobacteriota* was significantly higher in stressed mice than in non-stressed mice (Figure 2B). Furthermore, 14 genera showed significant differences in abundance between stressed and non-stressed mice: the abundance of four genera (*Parasutterella* and *Rikenellaceae_RC9_gut_group* being the most significant) was increased and that of the remaining 10 (*Bacteroides*, *Alistipes*, and *Lactobacillus* being the most significant) was decreased (Figures 2C,D). Moreover, a significant decline in diversity (Chao1 index) was observed in the gut microbiota of stressed mice (Figure 2E). We then performed linear discriminant analysis effect size (LEfSe) analysis to identify bacteria phylotypes that were differentially altered. As highlighted by the LEfSe plot and cladogram (Figures 2F,G), 62 taxa (30 for the stressed group and 32 for the control group) were identified as potential microbial markers. Overall, these findings indicated that psychological stress significantly impacted gut microbiota.

### 3.3. FMT of “affective disorder microbiota” increased stress sensitivity

**Figure 3:**
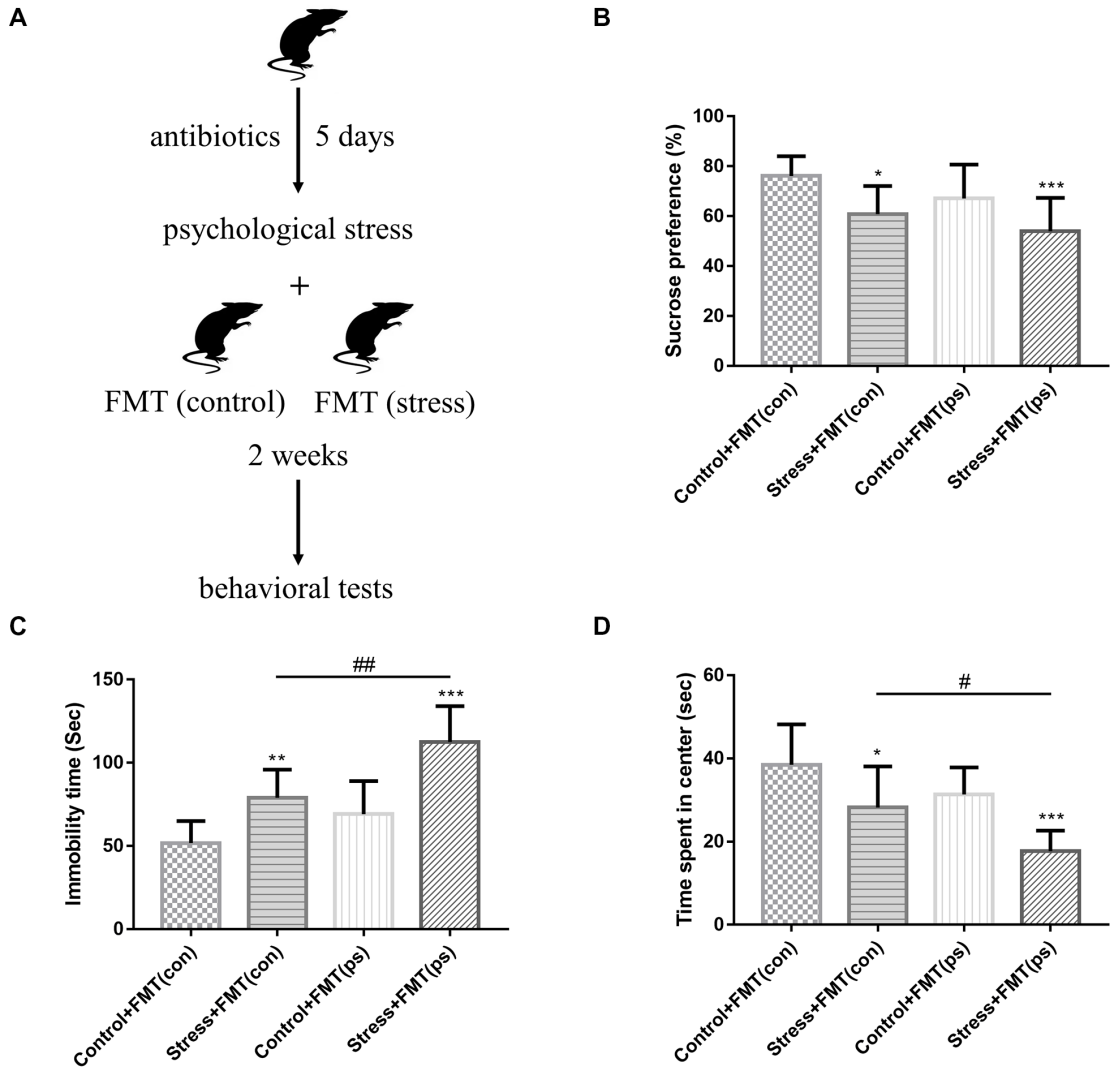
Effects of FMT of “affective disorder microbiota” on stress sensitivity. **(A)** Experimental design. **(B)** Psychological stress with FMT decreased the sucrose preference [*F*_(1,36)_ = 4.515, *p* = 0.0405; *F*_(1,36)_ = 14.6, *p* = 0.0005, respectively]. FMT of “affective disorder microbiota” increased the already high immobility time [two-way ANOVA FMT effect: *F*_(1,36)_ = 19.58, *p* < 0.0001] **(C)**, and exacerbated the decreased time mice spent in the center of the open field [two-way ANOVA FMT effect: *F*_(1,36)_ = 11.59, *p* = 0.0016] **(D)**. Values represent mean ± SD; *n* = 10/group. Two-way analysis of variance with Tukey’s post-hoc test was used to compare differences (*p < 0.05; **p < 0.01, ***p < 0.001 vs. the control group; # *p* < 0.05, ## *p* < 0.01).

To investigate whether changes in gut microbiome contribute to the pathogenesis of affective disorder in response to psychological stress, FMT of “affective disorder microbiota” derived from psychologically stressed mice and FMT of “normal microbiota” derived from non-stressed mice were performed (Figure 3A). Mice transplanted with microbiota were also sham-treated or subjected to psychological stress. SPT, FST, and OFT were conducted the day after FMT and psychological stress treatment. FST results showed that compared to transplantation of “normal microbiota,” transplantation of “affective disorder microbiota” further increased the already high immobility time (Figure 3C). OFT findings revealed that relative to transplantation of “normal microbiota,” transplantation of “affective disorder microbiota” exacerbated the decreased time mice spent in the center of the open field (Figure 3D). With regard to SPT, stressed mice transplanted with either “affective disorder microbiota” or “normal microbiota” exhibited a decreased preference for sucrose, and the decrease was more obvious in mice transplanted with “affective disorder microbiota,” but there was no statistical difference between the two stress + FMT groups (Figure 3B).

It is notable that non-stressed mice transplanted with “affective disorder microbiota” did not show a significant change in behavioral performance relative to those transplanted with “normal microbiota.” These data suggested that FMT of “affective disorder microbiota” derived from psychologically stressed mice resulted in increased stress sensitivity.

### 3.4. Untargeted metabolomics analyses

**Figure 4:**
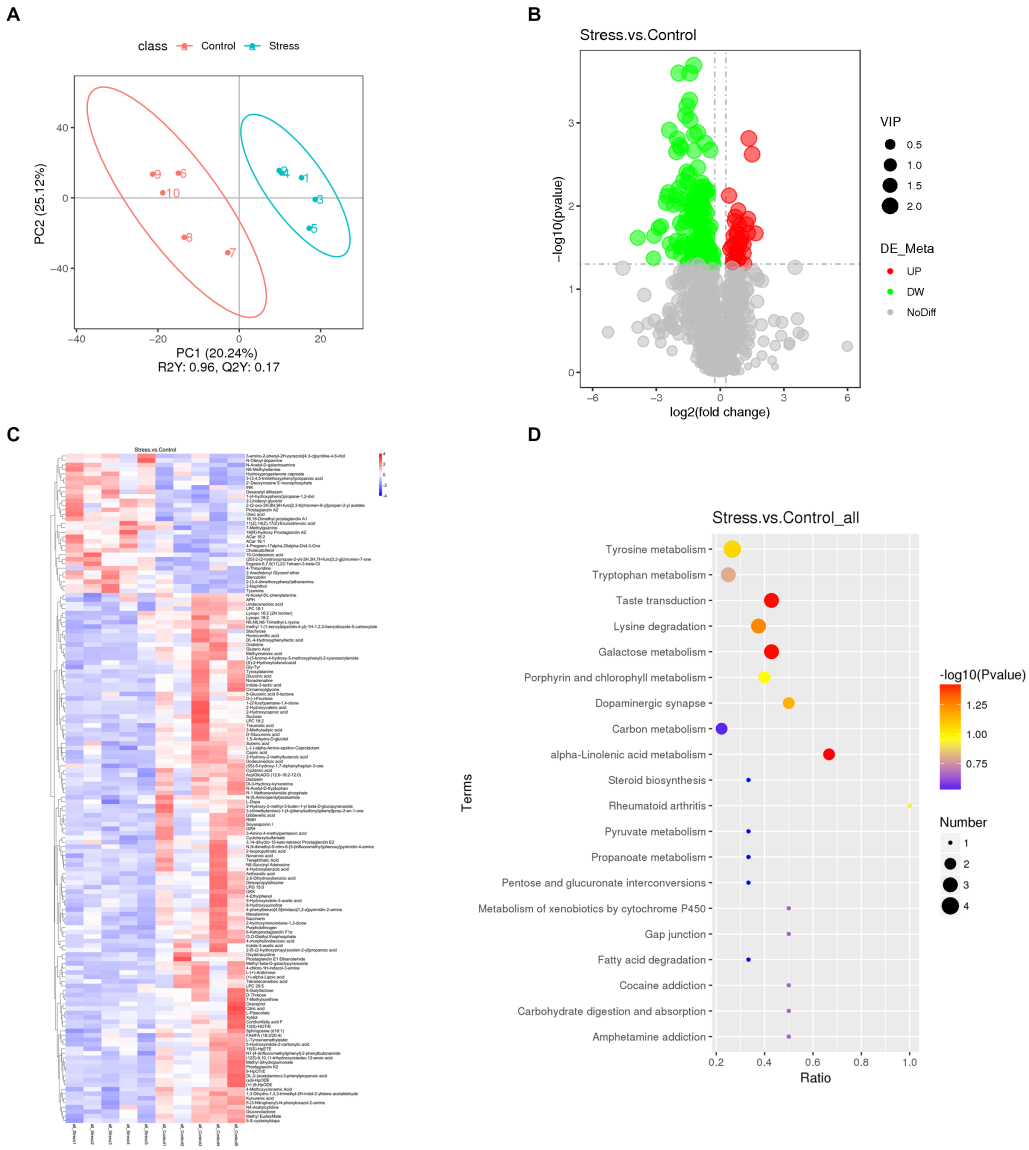
Fecal metabolomics analysis revealed significant alterations in metabolites between stressed and non-stressed mice (*n* = 5/group). **(A)** PLS-DA plot revealed metabolites with significant changes between the groups. **(B)** A volcano plot showing the overall distribution of differentially expressed metabolites (118 were down-and 31 were upregulated). **(C)** A heatmap showing details of differential metabolites. **(D)** KEGG pathway analysis based on significantly altered metabolites. Dot color represents *P*, and dot size represents the number of differential metabolites in the corresponding pathway.

Considering the key role of microbial metabolites on gut microbiome modulation and host pathophysiology, we examined the effects of psychological stress on metabolites due to microbial changes. PLS-DA showed that stressed and non-stressed mice exhibited unique metabolite profiles (Figure 4A). Overall, >1,000 gut metabolites were identified, of which 149 were differentially expressed: 118 were down-and 31 were upregulated (Figure 4B). These have been listed and clustered in Figure 4C. KEGG pathway analysis indicated that differential metabolites were mainly associated with α-linolenic acid metabolism, taste transduction, and galactose metabolism (Figure 4D). These findings showed that mice exposed to psychological stress showed substantial differences in fecal metabolite profiles, which seem to contribute to the development of affective disorders.

### 3.5. Correlation analysis

**Figure 5:**
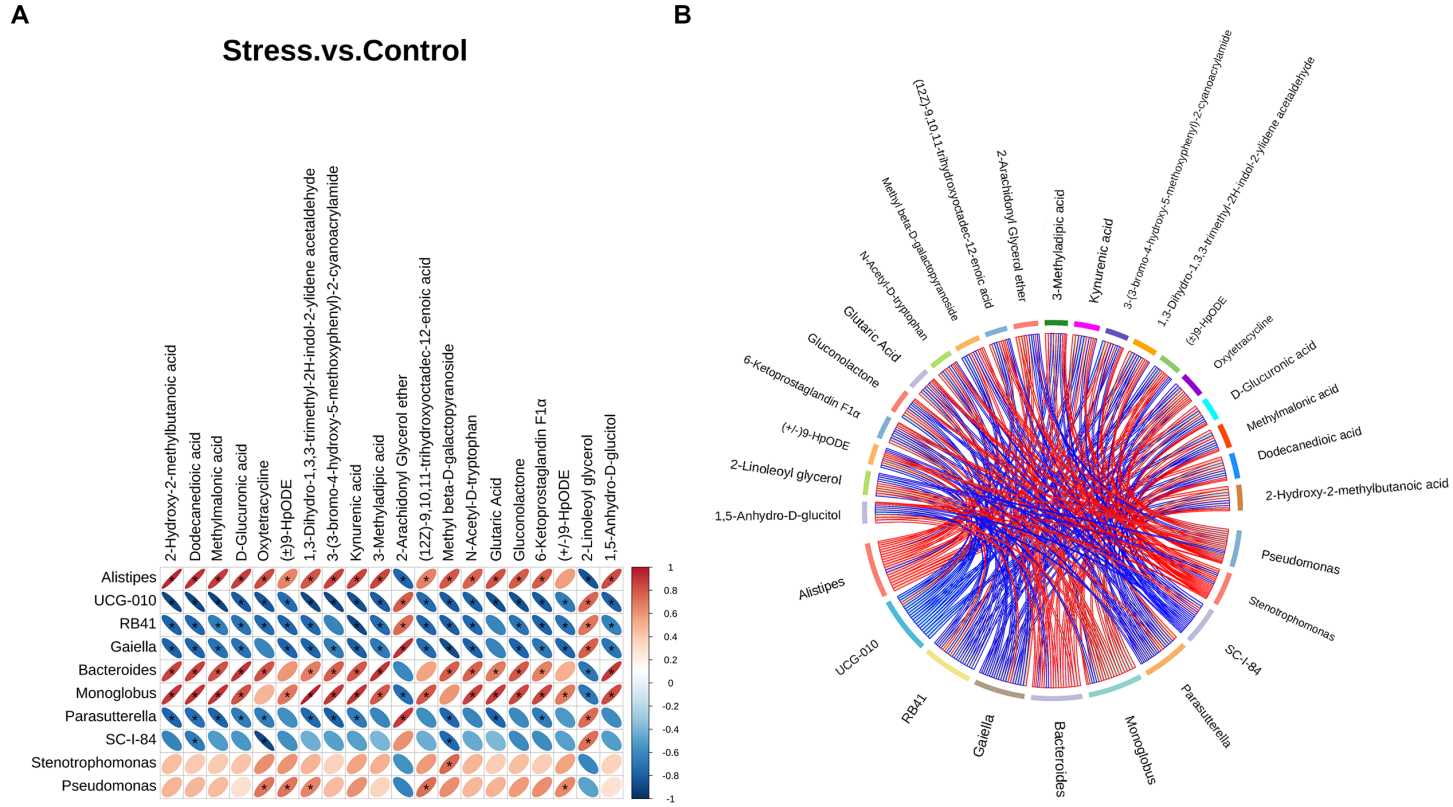
Correlation analysis between the top 10 differential bacteria at the genus level and top 20 differential metabolites. **(A)** Heatmap correlation analysis. The horizontal direction represents different bacteria, the vertical direction represents different metabolites, and the legend on the right shows the correlation coefficient. Red indicates positive correlation and blue indicates negative correlation; asterisk (*) indicates statistical significance (*p* < 0.05). **(B)** Chord diagram. Nodes represent different genera and metabolites. Chord width indicates correlation strength. Chord border color indicates correlation, with red indicating positive and blue indicating negative correlation.

We performed Spearman correlation analysis to evaluate the relationship between differential bacteria at the genus level and differential metabolites. Several metabolites showed a strong relationship with key differential gut microbiota. The top 10 genera and top 20 metabolites are listed and shown as a heatmap and chord diagram in Figures 5A,B, respectively. Among the bacteria genera that differed significantly from the 16S rRNA analysis, *Alistipes* and *Bacteroides* were mainly positively correlated while *Parasutterella* was mainly negatively correlated with several metabolites, such as 2-hydroxy-2-methylbutanoic acid, dodecanedioic acid, and kynurenic acid.

## 4. Discussion

Physical and psychological stress have been reported to negatively affect emotional behaviors (Shin and Liberzon, 2010; Li et al., 2016; Sun et al., 2018); moreover, different methods of stimulation exert different effects on animals (Haleem et al., 2014). In this study, we established a pure psychological stress model using a communication box wherein mice were exposed to chronic fear. SPT and FST results indicated that psychologically stressed mice exhibited depression-like behavior, and OFT results showed that they exhibited anxiety-like behavior. In addition, compared with non-stressed mice, stressed mice experienced weight loss. These data concur with the results of a previous study (Geng et al., 2019), validating that psychological stress induces affective disorders in mice.

Gut microbiota reportedly influence brain function and behavior via the “microbiota–gut–brain” axis (Bercik et al., 2011; Diaz Heijtz et al., 2011; Cryan and Dinan, 2012), and changes in gut microbiota composition may eventually contribute to the pathogenesis of stress-related affective disorders. Previous studies reported that in comparison to healthy controls, patients with depression showed altered microbiota composition and lower microbiota diversity (Barandouzi et al., 2020); FMT from patients with depression to microbiota-depleted rats induced anhedonia and anxiety-like behavior (Kelly et al., 2016). Another study found that chronic unpredictable mild stress promoted anxiety-and depression-like behavior in mice, which was associated with modulations in gut microbiota composition; besides, mice colonized with gut microbiota from stressed mice showed similar behaviors (Li et al., 2019). In this study, even we found that exposing mice to pure psychological stress significantly impacted gut microbiota composition, characterized by increased abundance of four genera and decreased abundance of 10 genera. Moreover, the transplantation of “affective disorder microbiota” exacerbated affective disorders induced by psychological stress as compared with the transplantation of “normal microbiota.” It is notable that non-stressed mice transplanted with “affective disorder microbiota” did not show any significant changes in behavioral performance, which is different from the findings of previous studies. In the earlier studies, colonizing germ-free mice with “depression microbiota” derived from individuals with major depressive disorder was found to induce depression-like behavior (Zheng et al., 2016); microbiome transplants from social defeat stress vulnerable rats were sufficient to recapitulate specific aspects of stress vulnerability including depression-like behaviors, although the authors did not observe increased anxiety-like behaviors of rats that received microbiota from stress vulnerable rats (Pearson-Leary et al., 2020). These contradictory findings may be due to differences in stress stimulation models where the “affective disorder microbiota” derived from. Our results suggest that psychological stress experience itself is required to produce changes in affective disorder behaviors, nevertheless, FMT of “affective disorder microbiota” derived from psychologically stressed mice resulted in increased stress sensitivity.

Microbial metabolites evidently facilitate gut–brain communication and behavior regulation (Banfi et al., 2021; Rosa et al., 2022). Considering that psychological stress influenced the abundance of specific gut microbiota, we next sought to examine changes in bacteria-derived metabolites. Our results indicated that stressed and non-stressed mice exhibited distinct metabolite profiles. Psychologically stressed mice were characterized by obvious disturbances in α-linolenic acid metabolism, taste transduction, and galactose metabolism. α-Linolenic acid, a major omega-3 fatty acid in animals and humans, is crucial for several neurocognitive functions. Altered omega-3 fatty acid levels reportedly play a role in reduced resistance to stress and mood disorders (Hennebelle et al., 2012). Taste transduction pathway is also involved in emotional regulation; Dmitrzak-Weglarz et al. observed that in women with unipolar depression, taste transduction pathway was downregulated (Dmitrzak-Weglarz et al., 2021). Galactose is crucial for human metabolism; in addition to its broad role in human physiology, galactose metabolism has been reported as beneficial in several diseases, particularly those affecting brain functions (Salkovic-Petrisic et al., 2014; Coelho et al., 2015). These three biological processes seem to be closely related to affective disorders caused by psychological stress. In addition, we also found tryptophan metabolism was regulated differently in stressed mice compared to control group. Tryptophan and its metabolites, such as serotonin, and other catabolites, such as kynurenine and its metabolites, have neuroactive properties, affecting the development of psychiatric diseases (Dantzer, 2017; Davidson et al., 2022). Recent studies have shown that gut microbiota could shape tryptophan metabolic pathways in multiple ways, via direct or indirect mechanisms, modulating host physiology and behavior, including functioning of immune system, gastrointestinal tract, metabolic processes, as well as neurodevelopment, anxiety and depressive behavior (Agus et al., 2018; Gheorghe et al., 2019). Further studies are warranted on this topic.

We also found that various metabolites showed a strong relationship with key differential gut microbiota at the genus level. Among the genera showing significant differences, *Alistipes* and *Bacteroides* were mainly positively correlated while *Parasutterella* was mainly negatively correlated with several metabolites, mainly organic acids, such as dodecanedioic acid and kynurenic acid. *Alistipes* and *Bacteroides* have been implicated in tryptophan metabolism (Liang et al., 2018; Parker et al., 2020), may contribute to the abnormal metabolism of kynurenic acid and tryptophan derivative N-Acetyl-D-tryptophan. Dodecanedioic acid is an even-number medium-chain dicarboxylic acids, with characteristics intermediating between glucose and fatty acids, and its decrease might be due to the reduction of substrate metabolism by gut microbiota (Guo et al., 2022). Therefore, the fatty acids and tryptophan metabolism might be involved in affective disorder development during bacterial translocation.

This study also has limitations. Our findings suggest that dysbiosis of the gut microbiome contributes to the development of affective disorders in response to psychological stress, but direct evidence that alteration of the gut metabolome is involved has not been provided. More importantly, it remains to be investigated which metabolites play the most important roles in improving affective disorder behaviors under psychological stress. We plan to focus on this in future research.

To summarize, psychological stress induced anxiety-and depression-like behaviors in mice. Further, psychological stress altered gut microbial diversity and metabolite profiles, and some core differential bacterial genera showed strong correlation with several differential metabolites. Finally, FMT of “affective disorder microbiota” derived from psychologically stressed mice resulted in increased stress sensitivity. Overall, these data suggest that alterations in gut microbiota can be involved in the development of affective disorders in response to psychological stress.

## Data availability statement

The datasets presented in this study can be found in online repositories. The name of the repository and accession number can be found below: NCBI Sequence Read Archive; PRJNA926785.

## Ethics statement

The animal study was reviewed and approved by the Institutional Animal Care and Use Committee of Gannan Medical University.

## Author contributions

YL conceived and designed the experiments. YZ, JZ, JW, and QZ performed the experiments. YZ and JZ helped to analyze and interpret the data. YL and CC drafted the manuscript. All authors reviewed and approved the final manuscript.

## Funding

This study was supported by grants from the National Natural Science Foundation of China (81960341) and the Natural Science Foundation of Jiangxi, China (20202BAB206032).

## Conflict of interest

The authors declare that the research was conducted in the absence of any commercial or financial relationships that could be construed as a potential conflict of interest.

## Publisher’s note

All claims expressed in this article are solely those of the authors and do not necessarily represent those of their affiliated organizations, or those of the publisher, the editors and the reviewers. Any product that may be evaluated in this article, or claim that may be made by its manufacturer, is not guaranteed or endorsed by the publisher.
